# Humoral Immunity to Primary Smallpox Vaccination: Impact of Childhood versus Adult Immunization on Vaccinia Vector Vaccine Development in Military Populations

**DOI:** 10.1371/journal.pone.0169247

**Published:** 2017-01-03

**Authors:** Bonnie M. Slike, Matthew Creegan, Mary Marovich, Viseth Ngauy

**Affiliations:** 1 U.S. Military HIV Research Program, Silver Spring, Maryland, United States of America; 2 The Henry M. Jackson for the Advancement of Military Medicine, Bethesda, Maryland, United States of America; 3 Division of AIDS, National Institute of Allergy and Infectious Diseases, Bethesda, Maryland, United States of America; 4 Tripler Army Medical Center, Honolulu, Hawaii, United States of America; Imperial College London, UNITED KINGDOM

## Abstract

Modified Vaccinia virus has been shown to be a safe and immunogenic vector platform for delivery of HIV vaccines. Use of this vector is of particular importance to the military, with the implementation of a large scale smallpox vaccination campaign in 2002 in active duty and key civilian personnel in response to potential bioterrorist activities. Humoral immunity to smallpox vaccination was previously shown to be long lasting (up to 75 years) and protective. However, using vaccinia-vectored vaccine delivery for other diseases on a background of anti-vector antibodies (i.e. pre-existing immunity) may limit their use as a vaccine platform, especially in the military. In this pilot study, we examined the durability of vaccinia antibody responses in adult primary vaccinees in a healthy military population using a standard ELISA assay and a novel dendritic cell neutralization assay. We found binding and neutralizing antibody (NAb) responses to vaccinia waned after 5–10 years in a group of 475 active duty military, born after 1972, who were vaccinated as adults with Dryvax®. These responses decreased from a geometric mean titer (GMT) of 250 to baseline (<20) after 10–20 years post vaccination. This contrasted with a comparator group of adults, ages 35–49, who were vaccinated with Dryvax® as children. In the childhood vaccinees, titers persisted for >30 years with a GMT of 210 (range 112–3234). This data suggests limited durability of antibody responses in adult vaccinees compared to those vaccinated in childhood and further that adult vaccinia recipients may benefit similarly from receipt of a vaccinia based vaccine as those who are vaccinia naïve. Our findings may have implications for the smallpox vaccination schedule and support the ongoing development of this promising viral vector in a military vaccination program.

## Introduction

The most successful vaccination campaign in human history used a vaccinia virus which led to the eradication of smallpox in 1980. In recent years, viral vectors have shown promise as vaccine delivery vehicles for various diseases. These vaccinia based recombinant vectors are popular because they are large stable DNA viruses that can accommodate extra genetic information. However, pre-existing immunity directed against the viral vector may diminish the elicitation of protective immune responses to the vaccine inserts and thereby limit their use [1, 2]. Scientists have employed various tactics to circumvent this obstacle by using alternate routes of administration (e.g. mucosal immunization), exploiting less common viral vectors (e.g. avipoxviruses, rare adenovirus serotypes, alphaviruses), or by priming with DNA prior to the viral vector boost in individuals with pre-existing immunity [3–7] to improve responses to the inserts.

Modified Vaccinia Ankara (MVA), a highly attenuated form of vaccinia, is a well-studied viral vector, with a large payload capacity, an excellent safety profile that targets antigen-presenting dendritic cells (DCs) and induces cell-mediated immunity [8–14]. As HIV vaccination strategies employing MVA continue to be explored, concerns regarding its generalizability to individuals with baseline immunity to vaccinia, such as military personnel and emergency responders, may impede its development. The presence of pre-existing immunity to vaccinia as a result of recent smallpox vaccination is of particular concern to the military in the development of a vaccinia-based vectored vaccine. In the United States, routine smallpox vaccination ceased in the early 1970’s in the civilian population. The military continued to vaccinate for smallpox at entry into active service until approximately 1991. Vaccination was discontinued until 2002, when it was re-initiated using Dryvax® for military and key civilian personnel in response to a potential terrorist threat. Dryvax® is a lyophilized, live-virus preparation derived from the New York City Board of Health vaccinia strain prepared from calf lymph [15]. On April 1, 2008, the Dryvax® vaccine was discontinued and replaced with ACAM2000™, a lyophilized, live vaccinia virus preparation derived from plaque purification cloning from Dryvax® and grown in African Green Monkey kidney (Vero) cells [16]. To date, over 2.4 million operational forces and health care workers have been vaccinated since December 2002 [17].

Prior reports demonstrate long-lasting vaccinia immunity after childhood vaccination [18–20]. Data in mice and humans suggest pre-existing anti-vaccinia immunity may limit the effectiveness of this viral vector for use as a vaccine candidate due to the continued presence of neutralizing antibodies [21, 22]. In a study by Rooney et al using a mouse model, they showed when animals were immunized with a vaccinia vector expressing the gD glycoprotein component (vaccinia/gD) of the herpes simplex virus 1 (HSV1) and given a subsequent booster dose 3 months later they generated an anamnestic response that substantially increased the production of HSV1 neutralizing antibodies while receipt of a vaccinia vector with a non-HSV insert showed a reduction in neutralizing antibody titers to HSV1 [22].

A variety of immunization strategies have been used to elicit the desired immune response while mitigating the effects of prior circulating anti-vaccinia antibodies. Results of a clinical trial conducted in Tanzania showed the vector efficiently induced HIV-1 antigen specific immune responses when used in a DNA prime-MVA boost combination in subjects with pre-existing immunity [23]. A more recent study by the HIV Vaccines Trials Network (HVTN205, NCT00820846) in healthy individuals, regardless of pre-existing immunity, comparing a DNA prime-MVA boost versus an MVA only schema showed both regimens elicited durable antibody and cell-mediated immune responses despite the development of anti-vaccinia antibodies with repetitive boosting [24]. Based on these promising results, this pilot study explores the durability of vaccinia antibodies after smallpox vaccination with Dryvax® or ACAM2000™, and how pre-existing humoral immunity to vaccinia affects entry and transgene expression of MVA-vectored vaccines in a primary human DC infection model in a unique and well characterized cohort of adult primary vaccinees.

## Materials and Methods

### Study Design

This study was approved by the Uniform Services University of Health Sciences (FWA 00001628; DoD Assurance P60001, study # IDCRP-026) and the Walter Reed Army Institute of Research Institutional Review Board (study# RV 260/WRAIR #1537). The study received an exempt determination from both IRBs and waiver of informed consent as investigators received aggregate de-identified serum samples from the DoD Serum Repository (DoDSR) and had no access to private health information. The DoDSR was established in 1989 for the purpose of storing leftover serum following mandatory HIV and operational deployment testing within the active and reserve components. Access to the sera and relevant demographic, occupational, and medical data is available to DoD investigators to support the conduct of military relevant research. The DoDSR maintains a link between the sera and the military member that allows access to demographic data such as year of birth, gender, race (self-reported), immunization records, and ICD-9 diagnosis codes (e.g. HIV serostatus). De-identification of serum samples was performed by the DoDSR.

**Fig 1:**
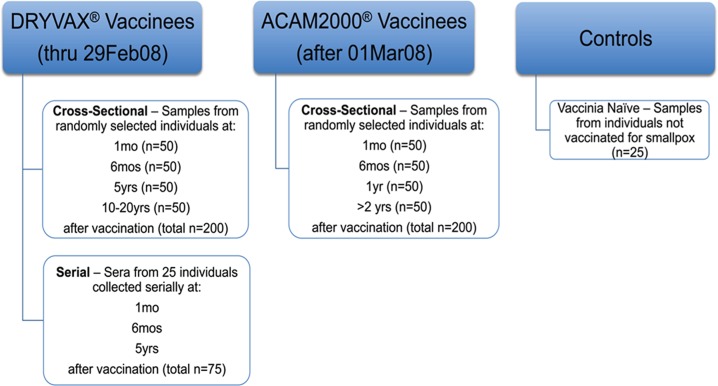
Study design. Sera from individuals were randomly selected by DoDSR staff based on the following inclusion criteria: the sample must be banked sera stored in the DoDSR, with available specimens at the requisite time points and with full documentation of smallpox immunization on record for the donor. Samples were excluded if the smallpox vaccination status was unknown, the sample was positive for HIV infection, or there was inadequate sera volume.

A total of 475 serum specimens from healthy individuals with known smallpox vaccination at specific time points and 25 vaccinia naïve controls were obtained from the DoDSR (Fig 1). Sera from military personnel vaccinated with either Dryvax® or ACAM2000™ were obtained at 4 time points post-vaccination. As a comparator, 25 individuals with longitudinal sera available at 3 corresponding time points were studied to address inter-individual variability in response over time. Anti-vaccinia rabbit IgG served as a positive control.

### Vaccinia Enzyme Linked Immunosorbent Assay (ELISA)

Sera obtained from the DoD serum repository were sent to V-Bio (St. Louis, MO), a validated laboratory used for vaccinia ELISA testing in human clinical trials. The vaccinia IgG ELISA procedure described previously was modified from Iaccono et al [25, 26]. Briefly, plates were coated with vaccinia antigen or negative (mock-infected) cell culture lysate. Serial 2-fold dilutions of sera were placed on both antigen-coated and mock-antigen coated wells and incubated for two-hours at 37°C. Following a standard washing procedure, horseradish peroxidase-conjugated anti-human IgG was added to the plate and the plates were again incubated for two hours at 37°C. After incubation, the plates were washed and ABTS substrate (Kirkegaard and Perry, Gaithersburg, MD) was added. Following a 30-minute incubation at room temperature, stopping solution (1% SDS) was added and the plates read at 405/492 nm dual wavelength. Linear regression plots were prepared and endpoint titers were determined based on an OD cut-off of 0.30 using UnitWin software. V-Bio provided vaccinia serology support for an MVA-based Phase I HIV-vaccine candidate clinical trial conducted at Rockville, MD (RV 158, NCT00376090).

### Preparation of Dendritic Cells (DCs)

Cryopreserved peripheral blood mononuclear cells (PBMCs) isolated from leukapheresis products of healthy donors, collected under an IRB-approved protocol were thawed for each DC preparation. PBMCs were adhered to tissue culture dishes for 60 min and after several RPMI washes, adherent cells were cultured in 10 mL complete media with 2x10^4^ U/mL rHu GM-CSF (Fisher Clinical Services, Allentown, PA) and 2 x10^4^ U/mL IL-4 (R&D Systems, Minneapolis, MN) for 7 days at 37°C, 5% CO_2_. On Day 6, 50 μL of Monocyte Conditioned Media mimic [MCM mimic, 15 μg/mL IL-6 (Peprotech Rocky Hill, NJ), 500 ƞg/mL IL-1β, 500 ƞg/mL TNF-α (Sigma St. Louis, MO) and 100 μg/mL PGE2 (Cayman Chemical, Ann Arbor, MI)] were added to mature the cells. The phenotype of all DCs was confirmed by flow cytometry. Specifically, DCs lack CD3, CD20 and CD14, but express high levels of HLA-DR and DC-SIGN. Mature cells additionally express CD25, CD83 and CD86 and reduced levels of DC-SIGN [27].

### MVA-GFP Neutralization Assay

A neutralization assay employing an attenuated, replication defective MVA carrying a green fluorescent protein reporter gene (MVA-GFP) was used as a readout system to determine anti-vaccinia virus neutralizing activity for each serum specimen at the various time points after immunization.

DCs were obtained from 5 different donors for use as targets. MVA-GFP constructs were obtained from the Laboratory of Viral Diseases, National Institute of Allergy and Infectious Diseases, NIH (generously provided by Bernie Moss, Patricia Earl). All sera samples were heat treated for 30 min at 56°C to inactivate complement. Sera were serially diluted in a volume of 50uL and pre-incubated with virus for 60 min at 37°C. The sera/virus complex was then added to 1x10^5^ DCs per well in a 96-well plate and incubated for 2 hours. Cells were then washed once, resuspended in complete media and incubated overnight at 37°C / 5% CO_2_. Cells were washed with phosphate-buffered saline then fixed in 2% paraformaldehyde. The percentage of MVA-infected DCs was evaluated by measuring GFP expression by flow cytometry on a LSR-II instrument (Becton Dickinson, Heidelberg, Germany) [28].

### Measurement of HIV Transgene Expression in MVA-CMDR

HIV transgene expression was determined by intracellular staining with anti-p24/gag (Dako, Glostrup,Denmark) and clade-specific anti-env mAbs and assessed by flow cytometry. Antigen expression was determined using human DCs as targets and the MVA-CMDR vector (MVA-Chiang Mai Double Recombinant, recombinant modified vaccinia Ankara viral vector expressing HIV-1 genes *env*/*gag*/*pol*) [9, 29]. Sera were heat inactivated and tested in the DC neutralization assay as above. Following the final incubation, cells were washed once with PBS then resuspended in cytofix/cytoperm solution (BD Biosciences) for 20 minutes at 4°C. Cells were washed and resuspended in perm/wash buffer (BD Biosciences) containing the appropriate concentration of mAb against the HIV transgene of interest (e.g. anti-p24/gag). Following staining for 15 minutes at 4°C, cells were washed twice and then resuspended with the matched fluorescently labeled secondary antibody for an additional 15 min. Secondary antibody was removed with washing and cells were resuspended in 2% paraformaldehyde and analyzed by flow cytometry.

### Statistics

Statistics were generated using Prism® software version 6, GraphPad Software Inc., La Jolla CA. Mann-Whitney and Spearman Rank Sum tests were applied as appropriate. Comparisons were deemed significant at p value cutoff of < 0.05.

## Results and Discussion

**Fig 2:**
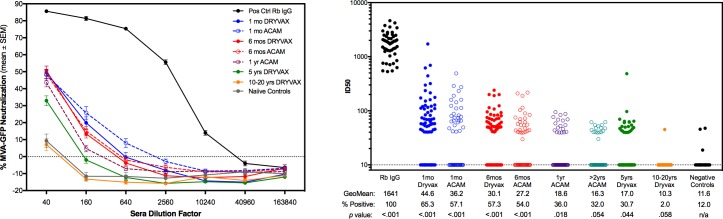
Neutralization of MVA-GFP infection of primary human dendritic cells. (A) Neutralization curves for each group are shown as the mean ± SEM of all serum samples at various dilutions. (B) Curves were analyzed by non-linear regression sigmoidal dose response curve fitting to determine the ID50 of each sample. Geometric mean and percent positive are indicated for each group. The p value was generated by Mann-Whitney unpaired t-test of each group compared to the negative control (vaccinia-naïve) group.

Neutralizing activity of sera from Dryvax® and ACAM2000™vaccinees was tested at 4 time points post-vaccination. Sera were tested at 4-fold dilutions for neutralization of infection of primary human DC with MVA-GFP (Fig 2A). Anti-vaccinia IgG from immunized rabbits served as a positive control (solid black line) and the mean neutralization from 25 vaccinia naïve donors was the negative control (solid grey line). Data were normalized to infection level in the no-sera control wells to account for inter-assay variability between 5 PBMC donors used for assays. The ID50s were calculated for each individual sample and are shown in Fig 2B. The percentage of positive responders (mean titer > 20) of samples tested is indicated below, as well as the p value indicating differences between groups and the response rate in the negative control group. Neutralization activity as measured by MVA-GFP expression, and geometric mean ID50 titers decreased with increasing time intervals from smallpox vaccination. Fewer individuals remained positive at 10–20 years after than 5 years after vaccination (2% versus ~30%).

On April 1, 2008, the Dryvax® vaccine was discontinued and replaced with ACAM2000™ for vaccination of military personnel and key emergency responders by the DoD. We tested sera using the same MVA-GFP neutralization assay at 4 time points post-ACAM2000™ vaccination for comparability (Fig 2B). Because ACAM2000™ has only been in use since 2008, long range downstream time points were not yet available at the time of study completion. Neutralization kinetics did not differ significantly between Dryvax® and ACAM2000™vaccinee sera (Fig 2B). Due to the short time for follow up after vaccination, definitive conclusions cannot be drawn regarding the differences in durability of vaccinia antibodies between Dryvax® and ACAM2000™.

**Fig 3:**
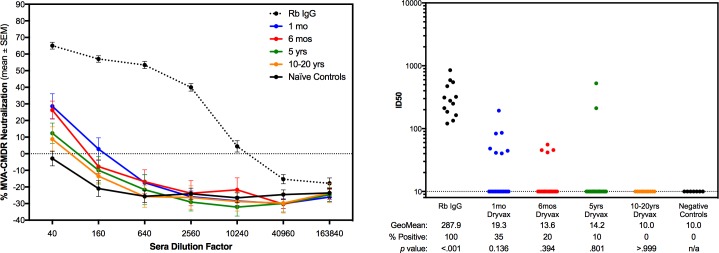
Neutralization of HIV transgene expression from an MVA-vectored Phase 1 vaccine. (A) Neutralization curves for a randomly selected subset (20%) of each of the testing groups are shown as the mean ± SEM of all samples at each sera dilution tested. (B) Curves were analyzed by non-linear regression sigmoidal dose response curve fitting to determine the ID50 of each sample. Geometric mean and percent positive are indicated for each group. The p value was generated by Mann-Whitney unpaired t-test of each group to the negative control (vaccinia-naïve) group.

Further characterization of this DC neutralization assay was performed on sera from a randomly selected subset of the Dryvax-inoculated samples tested above, using a second recombinant MVA-vectored HIV vaccine construct, MVA-CMDR. MVA-CMDR is a multigenic vaccine containing 3 HIV gene inserts: *env/gag/pol* derived from CRF01_AE isolates from Chiang Mai, Thailand (HIV-1 CM235 *env/CM240 gag/pol*) [9, 29]. This vaccine candidate was evaluated in an IRB approved Phase 1 study (RV 158) under FDA-IND #12267 conducted by the US Military HIV Research Program (MHRP) in Rockville, Maryland [30]. Twenty percent of the 50 cross-sectional samples at each time point of the Dryvax® sera and controls were tested for ability to neutralize this construct in the same DC-based system (Fig 3). Neutralization and inhibition of HIV transgene expression was determined using human DC obtained from healthy donors as targets for infection with MVA-CMDR. Cells were cultured and infected as with the MVA-GFP assay, and HIV transgene expression was determined by intracellular staining for HIV-p24/gag and assessed by flow cytometry. Expression and neutralization of HIV p24/gag data in DC were equivalent to that of GFP (1:1), thus it was only performed for the Dryvax® samples and not for the ACAM2000™ (Fig 3). As seen in the neutralization assay for MVA-GFP alone without transgene inserts, the neutralization activity of the sera also decreased with increased time from smallpox vaccination.

**Fig 4:**
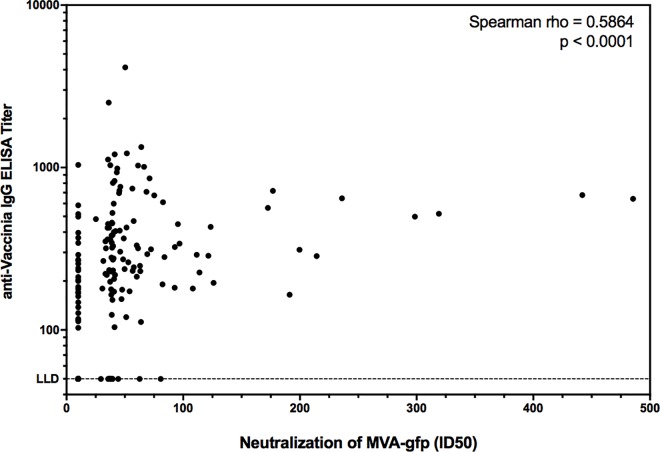
Correlation between anti-vaccinia ELISA and MVA-GFP neutralization. All Dryvax® samples tested in the MVA-GFP neutralization assay were also assessed in the traditional anti-vaccinia IgG ELISA. Correlation is shown for the 200 independent cross-sectional samples. Correlation was analyzed by Spearman ranked sum test. The lower limit of detection (LLD) for the ELISA is indicated.

As part of the screening process for MHRP-conducted clinical trials utilizing MVA-vectored candidate vaccines, participants undergo baseline testing for pre-existing vaccinia immunity using a validated anti-vaccinia ELISA assay (V-Bio, St. Louis MO). We employed this same test for samples from the Dryvax® groups within this study to determine the utility of the novel DC neutralization assay we developed. As shown in Fig 4, there was a robust correlation between anti-vaccinia IgG titer and MVA-GFP neutralization ID50 (Spearman rho = 0.5864, *p*<0.0001).

**Fig 5:**
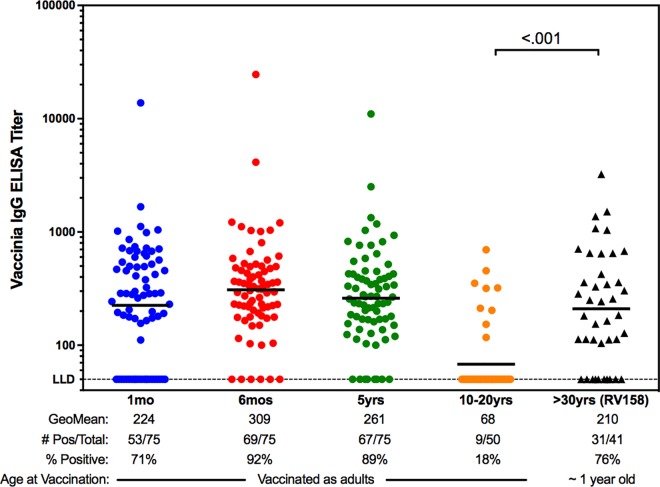
Influence of age at immunization on immunity duration. Anti-vaccinia ELISA titers are shown for the Dryvax® groups of the present study of military personnel vaccinated as adults in comparison to results from screening of civilian participants in a separate study (RV 158) who were known to be vaccinated with Dryvax® as children (right panel). The geometric mean titers and percent positive responders per group are indicated. Comparison between the most distal time points from vaccination were done via Mann-Whitney unpaired t-test.

As indicated earlier, MHRP conducted screening for vaccinia immune responses as a pre-requisite for any clinical study where an MVA-vectored candidate vaccine was to be tested. To that end, healthy, HIV-negative civilian volunteers ages 18–49 were screened for pre-existing vaccinia immunity for a recent Phase I clinical study, RV 158 [9]. The screening was assessed by ELISA at V-Bio Inc. (St. Louis, MO) using the same assay conducted for the present study. Forty-one individuals had pre-existing immunity and were excluded from participation in RV 158. In Fig 5, we compared the ELISA titers of these individuals with those from the Dryvax® groups of the present study. Individuals screened for RV158 [9] and found to have positive vaccinia titers were generally older (born before 1972), had a visible vaccination scar, and had received their smallpox vaccination during childhood.

In contrast to the near complete waning of response in the adult military vaccinees in the present study, the 41 civilians screened but excluded from the RV158 study participation due to positive anti-vaccinia antibody responses had GMTs of 210, with 76% of those screened found positive for circulating vaccinia binding antibodies (Fig 5). As civilians, these subjects were vaccinated in childhood with Dryvax® prior to 1970, indicating lasting humoral immunity of 30 plus years.

## Conclusions

Our findings in this adult military cohort differ from previous reports regarding long-lasting durability of anti-vaccinia antibody responses [18–20] and those of our comparator HIV vaccine trial volunteers who were vaccinated in childhood. There is little difference between the original Wyeth Dryvax® used in the 1950’s and 1960’s compared to the current one used by the DoD, so a possible alternate explanation for this incongruity is the age at vaccination (adult versus childhood). Most individuals vaccinated during the mass smallpox campaign were children, whereas, those vaccinated at entry into military service or for “force protection” measures were adults. Immunogenicity studies from two licensed vaccines (Pneumococcus and HPV) suggest age at vaccination influences the quantity and quality of antibody responses [30, 31]. Younger age at vaccination was associated with a better antibody response, quantitatively and functionally. In a meta-analysis of 24 published studies, Fisman et al evaluated the response to hepatitis B vaccine relative to age. Pooled study results showed a significantly increased risk of nonresponse to hepatitis B vaccine among older individuals (relative risk [RR], 1.76; 95% confidence interval [CI], 1.48–2.10), with older individuals defined as young as 30 years of age [32]. Additionally, children vaccinated during the original eradication campaign may have been more likely to be exposed to other contemporaneously vaccinated children, thereby getting “boosted” through repeat exposure to uncovered, infectious, open wounds at vaccination sites. Understanding the relatively rapid decline of humoral responses to smallpox in this unique cohort is beyond the scope of this pilot study, potentially requiring prospective trials to find a mechanism, but there is likely an immunologic basis underpinning age specific humoral anti-viral immunity.

A major priority for HIV vaccine development is the elicitation of broadly neutralizing antibodies (bNAbs). This remains an elusive goal but recent progress in this area and the study of infants may help further our understanding. In the last 5 years, technological advances have enabled the isolation of a variety of different families of bNAbs, each directed towards distinct targets on HIV-1 envelope, from a subset of HIV infected individuals [33]. However, these bNAbs are rather unique showing high levels of somatic hypermutation, long heavy chain complementarity determining region 3 (CDRH3), tendency towards autoreactivity, and require years to develop [33]. Many of these features can be limited by host tolerance controls. Notably, one recent report describes an infected infant who developed bNAbs within the first year of life and suggests that the immature immune system may be more amenable to developing protective antibodies and in much shorter time frames thus informing the vaccine field at large [34]. Ultimately, targeting younger age bands for vaccines, while required to prevent childhood diseases, may prove to be a winning strategy for threats encountered later in life as well.

There are several limitations to this study. Sera from military personnel were collected and stored as part of routine clinical or operational assessment visits (e.g. HIV testing prior to deployment). Storage and transport conditions of the serum specimens prior to arrival at the repository is variable and samples may experience delays and temperature extremes (heat). As the samples are de-identified, we are unable to obtain information on the health status of the individuals providing the samples, the quality of the “take” after vaccination, receipt of concomitant vaccines, prior undocumented smallpox vaccination and other undiagnosed immune suppressive conditions other than HIV status. However, based on the uniformity of the data and the medical screening conducted prior to administration of the smallpox vaccine to ensure the individual is healthy and does not have a contraindication to receipt of a live viral vaccine (eczema, cancer, immunosuppressive drugs, etc) we do not suspect the early decline seen in our study is due to degradation of the samples or to variability in the sample population.

In an adult military primary vaccinee population, humoral responses to smallpox vaccination (to Dryvax® and ACAM2000™) do not persist as long as previously reported in the civilian population that was vaccinated during childhood. Our findings were confirmed by a binding assay performed at a reference laboratory and by a functional neutralization assay using DCs as targets and MVA as a vector with two different readout modalities (GFP expression and HIV p24 transgene expression). These data suggest first that the age of primary vaccination influences the durability of humoral immunity and second that MVA encoding a transgene product can infect target DCs and efficiently express transgene products. Our findings suggest pox vector based vaccines targeting other diseases may be used in the military population with recent recipients of the smallpox vaccine benefiting as well as vaccinia naïve individuals. This finding also has implications on the timing of the smallpox vaccination schedule and supports the ongoing development of this promising viral vector in a military and civilian vaccination program.
